# Effect of COVID-19 pandemic on inpatient service utilization and patient outcomes in Uganda

**DOI:** 10.1038/s41598-023-36877-9

**Published:** 2023-06-15

**Authors:** Irene Andia-Biraro, Joseph Baruch Baluku, Ronald Olum, Felix Bongomin, Andrew Peter Kyazze, Sandra Ninsiima, Phillip Ssekamatte, Davis Kibirige, Samuel Biraro, Emmanuel Seremba, Charles Kabugo

**Affiliations:** 1 Makerere University College of Health Sciences, Kampala, Uganda; 2 MRC/UVRI & LSHTM Uganda Research Unit, Entebbe, Uganda; 3 Kiruddu National Referral Hospital, Kampala, Uganda; 4 Makerere University Lung Institute, PO Box 26343, Kampala, Uganda; 5 Department of Medicine, St Francis Hospital Nsambya, Kampala, Uganda; 6 Department of Medical Microbiology & Immunology, Faculty of Medicine, Gulu University, Gulu, Uganda; 7 Uganda Martyrs Hospital, Lubaga, Uganda; 8 Clockworks Research Company Limited, Kampala, Uganda

**Keywords:** Health care, Risk factors, Signs and symptoms

## Abstract

COVID-19 has had devastating effects on health systems but reports from sub-Saharan Africa are few. We compared inpatient admissions, diagnostic tests performed, clinical characteristics and inpatient mortality before and during the COVID-19 pandemic at an urban tertiary facility in Uganda. We conducted a retrospective chart review of patients admitted at Kiruddu National Referral Hospital in Uganda between January–July 2019 (before the pandemic) and January–July 2020 (during the pandemic). Of 3749 inpatients, 2014 (53.7%) were female, and 1582 (42.2%) had HIV. There was a 6.1% decline in admissions from 1932 in 2019 to 1817 in 2020. There were significantly fewer diagnostic tests performed in 2020 for malaria, tuberculosis, and diabetes. Overall, 649 (17.3%) patients died. Patients admitted during the COVID-19 pandemic (adjusted odds ratio [aOR] 1.2, 95% confidence interval [CI] 1.04–1.5, p = 0.018), patients aged ≥ 60 years (aOR 1.6, 95% CI 1.2–2.1, p = 0.001), HIV co-infected (aOR 1.5, 95% CI 1.2–1.9, p < 0.001), and those admitted as referrals (aOR 1.5, 95% CI 1.2–1.9, p < 0.001) had higher odds of dying. The COVID-19 pandemic disrupted inpatient service utilization and was associated with inpatient mortality. Policy makers need to build resilience in health systems in Africa to cope with future pandemics.

## Introduction

The corona virus of 2019 (COVID-19) has been the leading cause of death from an infectious agent since 2020. The lockdowns that were instituted also had a devastating impact on health care provision. As of 1st June 2022 there have been more than 527 million confirmed cases and 6.2 million deaths globally^1^. Nationwide and local social lockdowns, where persons are required to stay indoors to avoid inter-personal contact outside one’s family members, were instituted in several countries in a bid to prevent the spread of the disease^2^. During such lockdowns, public assembly was restricted and movement of the public was prohibited^2^. However, lockdown measures had the potential to affect access and utilisation of health services and result in a reduction child vaccination coverage^3^, an increase in maternal and child mortality^4^ and a rise malaria-related deaths^5^. Among people living with HIV (PLHIV), modelling showed that a 6-months interruption in antiretroviral therapy (ART) supply would result in a twofold rise in HIV-related deaths and maternal-to-child HIV transmission in sub-Saharan Africa^6^.

The overall utilisation of health services also suffered during the pandemic. A recent systematic review showed that the COVID-19 pandemic resulted in a 37% reduction in health service utilisation, including patient visits (42% reduction), admissions (28%), diagnostics (31%), and therapeutics (30%)^7^. However, there was no representation of studies from Africa in the systematic review. A study from Ethiopia reported an increase in childhood malnutrition and declines in detection of new HIV cases, cardiovascular disease diagnosis, cervical cancer screening and blood bank services^8^. Other studies from Africa suggest that the COVID-19 related lockdowns had negative effects on malaria, tuberculosis, HIV, reproductive health, and maternal-child health services and clinic appointments for chronic disease care^9^. There are few reports on the effect of the COVID-19 pandemic and its associated lockdowns on inpatient mortality in Africa.

In March 2020, Uganda instituted a nationwide lockdown that progressively banned private and public transport except for essential services and cargo^10^. Initial reports indicated a reduction in new HIV and malaria cases detected, fewer people were initiated on TB preventive therapy, there was a reduction in facility maternal deliveries and an increase in maternal mortality^11^. However, all-cause mortality at health facilities was reportedly level between 2019 (before the pandemic) and 2020 (early periods of the pandemic), although the data were drawn from health management information systems that had relatively low reporting rates^12^. In this study, the objective was to compare inpatient attendance, imaging and laboratory services accessed by inpatients, patient characteristics and inpatient mortality before and during the COVID -19 pandemic at an urban tertiary facility in Uganda.

## Materials and methods

### Study design, settings, and population

This was a retrospective longitudinal study conducted at Kiruddu National Referral Hospital (KNRH). KNRH has a 200-bed capacity and is one of four national referral hospitals in Uganda and located in Kampala, the capital city of Uganda. It is mandated to offer mainly specialized internal medicine and burns/reconstructive surgical services. The study population were patient files of adults admitted on the internal medicine units (cardiology, infectious diseases, pulmonology, nephrology, gastroenterology, hematology, and neurology) from January to July 2019 (before the pandemic) and from January to July 2020 (during the pandemic). We excluded patient files with > 20% missing data as this could cause misclassification bias. We conducted a census of all eligible files.

### Data collection

We conducted a census of all files of patients admitted during the periods under consideration. Files were retrieved from the hospital records office and consecutively reviewed using a data abstraction form. Data on patient demographics, reasons for admission, presenting complaints, baseline vital signs, comorbidities, diagnostic tests performed, and treatment outcomes were collected. Treatment outcomes are documented in the file by the attending physician upon discharge (or death) of the patient. As such the physician indicated whether the patient was cured, improved, unimproved, transferred and died.

### Data analysis

Data were exported to Microsoft Excel 2016 for cleaning and coding and imported to STATA 16.0 (Stata Corp LLC, College Station, Texas, USA) for formal analysis. First, numerical data was tested for normal distribution using Shapiro–Wilk test. Normally distributed numerical data were summarized as mean (standard deviation) whereas non-parametric numerical data as median (interquartile range). Categorical data were summarized as frequencies and percentages. At bivariate analysis, Chi-square test or Fisher’s exact test were used to assess the distribution of mortality across independent variables (sociodemographic and clinical characteristics). Simple logistic regression analysis was also used to assess the strength of these associations and presented as crude odds ratio at 95% confidence intervals. All independent variables with a p < 0.2 at bivariate analysis were used to construct multivariable logistic regression models in addition to variables that we deemed to have biological plausibility in influencing the risk of mortality. The goodness-of-fit was tested using Pearson goodness-of-fit test and the Hosmer–Lemeshow goodness-of-fit test and confusion matrix. The Hosmer–Lemeshow goodness-of-fit p-value was 0.748 and the confusion matrix showed, using the variables included in the multivariable logistic regression, the model correctly classified 82.54% of the mortality reported, both indicating that the model had a good fit. Results from the multivariable logistic regression model are presented as adjusted odds ratio at 95% confidence interval. At all levels of hypothesis testing, a p-value less than 0.05 were considered statistically significant.

### Ethics declarations

The study was approved by the ethics committee of the School of Medicine, College of Health Sciences, Makerere University (REC approval number 2020-170) and Uganda National Council for Science and Technology (HS1030ES approval number). The School of Medicine, College of Health Sciences, Makerere University ethics committee waived the need of participant consent since we used retrospective data. Data were de-identified by using codes on data abstraction forms instead of patient names. The authors assert that all procedures contributing to this work comply with the ethical standards of the relevant national and institutional committees on human experimentation and with the Helsinki Declaration of 1975, as revised in 2008. All methods and experimental protocols were carried out in accordance to the declaration of Helsinki.

## Results

**Figure 1:**
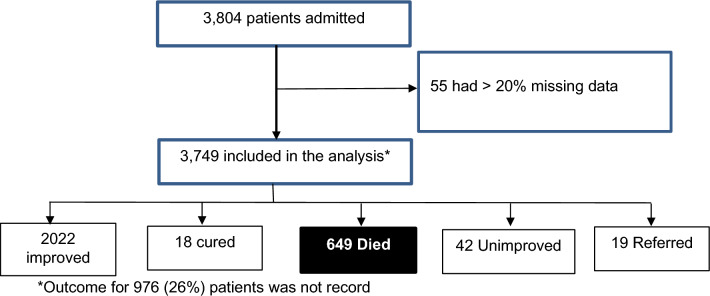
Study flow diagram.

A total of 3804 files of patients who were admitted in the in-patient department between January–July 2019 and January–July 2020 was reviewed. Of these files, 55 (1.4%) were excluded due missing data. Figure 1 shows the participant accrual process.

### Characteristics of patients

Table 1 shows the socio-demographic characteristics. Of 3749 patients, 2014 (53.7%) were female. More than half of the patients were aged more than 35 years old, with the elderly (60 + years) forming 21.3% of the patient population. Half of the patients were from the infectious disease unit (51.1%). The most frequent chronic diseases were HIV (42.2%), hypertension (18.0%) and diabetes mellitus (16.5%). About 20.6% and 9.6% of the patients had a history of alcohol use and smoking, respectively. The median duration of smoking and alcohol usage was 6.5 (IQR 3.5–20) years and 10 (5–20) years, respectively. About 7.3% (n = 275) had a history of both smoking and alcohol usage. More patients (51.6%) were admitted before the COVID-19 pandemic in 2019 (January–July 2019) than during the pandemic in 2020 (January–July 2020) indicating a 6.1% decline. By year of admission, patients differed by age category (p = 0.018), admission unit (p = 0.002), and region of origin in Uganda (p = 0.001). More patients with chronic obstructive pulmonary disease (COPD) were admitted in 2020 compared to 2019 (23 vs 11, p = 0.024).

**Table 1:** Socio-demographic characteristics of the participants.

Variables (N = 3749)	Overall: n (%)	2019: n (%)	2020: n (%)	p
Overall	3749.0	1933 (51.6)	1816 (48.4)	–
Age in years (median, IQR)	42 (30–56)	41 (30–56)	42 (30–56)	0.558
13–17 years	18 (0.5)	3 (0.2)	15 (0.8)	0.018
18–35 years	1347 (35.9)	711 (37.1)	636 (35.6)	
36–59 years	1549 (41.3)	790 (41.3)	759 (42.5)	
60 + years	787 (21)	410 (21.4)	377 (21.1)	
Sex				
Male	1735 (46.3)	865 (44.7)	870 (47.9)	0.053
Female	2014 (53.7)	1068 (55.3)	946 (52.1)	
Unit				
IDF	1024 (27.3)	540 (27.9)	484 (26.7)	0.002
IDM	893 (23.8)	433 (22.4)	460 (25.3)	
Pulmonology	482 (12.9)	273 (14.1)	209 (11.5)	
Cardiology	864 (23)	430 (22.2)	434 (23.9)	
Endocrine	466 (12.4)	253 (13.1)	213 (11.7)	
Others	20 (0.5)	4 (0.2)	16 (0.9)	
Region of origin				
Central	3293 (87.8)	1671 (87.9)	1622 (93.4)	0.001
West	152 (4.1)	88 (4.6)	64 (3.7)	
East	37 (1)	13 (0.7)	24 (1.4)	
North	204 (5.4)	128 (6.7)	76 (4.4)	
Religion				
Christian	2568 (68.5)	1407 (72.8)	1161 (63.9)	0.962
Muslim	685 (18.3)	376 (19.5)	309 (17)	
Unknown	496 (13.2)	150 (7.8)	346 (19.1)	
Patients with comorbidities				
HIV	1582 (42.2)	814 (42.1)	768 (42.3)	0.911
Hypertension	673 (18)	340 (17.6)	333 (18.3)	0.551
Diabetes mellitus	617 (16.5)	318 (16.5)	299 (16.5)	0.991
Cardiac disorder	320 (8.5)	158 (8.2)	162 (8.9)	0.413
Tuberculosis	319 (8.5)	154 (8)	165 (9.1)	0.220
Asthma	94 (2.5)	43 (2.2)	51 (2.8)	0.253
Kidney disease	41 (1.1)	18 (0.9)	23 (1.3)	0.324
Malignancy	19 (0.5)	6 (0.3)	13 (0.7)	0.081
COPD	34 (0.9)	11 (0.6)	23 (1.3)	0.024
History of smoking	361 (9.6)	187 (9.7)	174 (9.6)	0.923
History of alcohol usage	772 (20.6)	402 (20.8)	370 (20.4)	0.949
Purpose of visits				
Non-respiratory symptom	3609 (96.3)	1869 (96.7)	1740 (95.8)	0.158
Respiratory symptom	2143 (57.2)	1169 (60.5)	974 (53.6)	0.000
Referral	491 (13.1)	270 (14)	221 (12.2)	0.103
Need for oxygen	22 (0.6)	15 (0.8)	7 (0.4)	0.118
Others	174 (4.6)	93 (4.8)	81 (4.5)	0.610

*IDM* infectious disease (male) unit, *IDF* infectious disease (female) unit, *COPD* chronic obstructive pulmonary disease.

### Presenting symptoms and reason for admissions

A total of 491 patients (13.1%) were referrals from peripheral health facilities and some 22 (0.6%) patients needed oxygen on admission. Overall, the most frequent presenting symptoms were cough (43.2%), fever (36.7%), dyspnea (34.9%), general body weakness (27.5%) and headache (25.4%). About 12.4% and 5.1% presented with altered level of consciousness and convulsions, respectively. A lower proportion of patients were admitted with respiratory symptoms in 2020 compared to 2019 (45.5% vs 54.5%, p < 0.001). By year, patients presenting with cough (p < 0.001), sputum production (p = 0.001) and wheezing (p = 0.030) were significantly more in 2019 compared to 2020, whereas those presenting with fatigue (p = 0.018), altered level of consciousness (p = 0.001) and sore throat (p < 0.001) were significantly higher in 2020 than 2019. Table 2 summarizes the presenting complaints by year of admission.

**Table 2:** Presenting complaints of the patients.

Presenting complaints	Overall	2019: n (%)	2020: n (%)	p
Cough	1621 (43.2)	903 (46.7)	718 (39.5)	0.000
Fever	1376 (36.7)	733 (37.9)	643 (35.4)	0.111
Dyspnoea	1308 (34.9)	697 (36.1)	611 (33.6)	0.121
General body weakness	1030 (27.5)	546 (28.2)	484 (26.7)	0.274
Headache	951 (25.4)	495 (25.6)	456 (25.1)	0.726
Chest pain	872 (23.3)	461 (23.8)	411 (22.6)	0.378
Vomiting	860 (22.9)	459 (23.7)	401 (22.1)	0.266
Sputum production	697 (18.6)	400 (20.7)	297 (16.4)	0.001
Reduced appetite	680 (18.1)	335 (17.3)	345 (19)	0.186
Fatigue	524 (14)	245 (12.7)	279 (15.4)	0.018
Loose stool	470 (12.5)	259 (13.4)	211 (11.6)	0.100
Limb swelling	469 (12.5)	234 (12.1)	235 (12.9)	0.440
Altered level of consciousness	464 (12.4)	205 (10.6)	259 (14.3)	0.001
Visual disturbances	279 (7.4)	156 (8.1)	123 (6.8)	0.130
Urinary symptoms	253 (6.7)	125 (6.5)	128 (7)	0.478
Abdominal distension	210 (5.6)	107 (5.5)	103 (5.7)	0.856
Convulsions	191 (5.1)	90 (4.7)	101 (5.6)	0.208
Neck pain	172 (4.6)	96 (5)	76 (4.2)	0.253
Joint pain	158 (4.2)	82 (4.2)	76 (4.2)	0.930
Haemoptysis	119 (3.2)	65 (3.4)	54 (3)	0.497
Wheezing	96 (2.6)	60 (3.1)	36 (2)	0.030
Sore throat	25 (0.7)	4 (0.2)	21 (1.2)	0.000
Nasal congestion	16 (0.4)	5 (0.3)	11 (0.6)	0.103

### Diagnostic tests performed

Table 3 summarizes the 20 most common laboratory tests and imaging that were performed. There were significantly fewer tests performed in 2020 than 2019 for the following tests: complete blood count, renal function tests, electrolytes, liver function tests, urinalysis, sputum GeneXpert, blood smears for malaria, blood sugar, glycated hemoglobin, and cerebral spinal fluid analysis (all p < 0.05). Only the CD4/viral load tests were performed more in 2020 than 2019. The decrease was 20.5% in blood slides for malaria, 21.5% in sputum GeneXpert, 30.1% in glycated hemoglobin and 29.4% in blood glucose. Similarly, the decline was 57.5% in serum electrolytes, 46.5% in cerebral spinal fluid analyses, 26.2% in liver function tests, 22.5% in urinalysis, 21.8% in renal function tests, 12.1% in complete blood counts.

**Table 3:** Common diagnostics tests performed on patients admitted at the in-patient department of Kiruddu National Referral Hospital.

Diagnostic test	Overall: n (%)	2019: n (%)	2020: n (%)	p
Complete blood count	2992 (79.8)	1592 (82.4)	1400 (77.1)	0.041
Renal function tests	2433 (64.9)	1365 (70.6)	1068 (58.8)	0.000
Liver function tests	2136 (57)	1229 (63.6)	907 (49.9)	0.000
Electrolytes	925 (24.7)	649 (33.6)	276 (15.2)	0.000
Urinalysis	742 (19.8)	418 (21.6)	324 (17.8)	0.018
Sputum gene-Xpert	705 (18.8)	395 (20.4)	310 (17.1)	0.035
Blood smear for malaria	684 (18.2)	381 (19.7)	303 (16.7)	0.017
Serum cryptococcal antigen	680 (18.1)	365 (18.9)	315 (17.3)	0.222
CD4/viral load	513 (13.7)	247 (12.8)	266 (14.6)	0.034
Random/fasting blood sugar	493 (13.2)	289 (15)	204 (11.2)	0.003
Urine LAM for TB	466 (12.4)	235 (12.2)	231 (12.7)	0.341
HIV test	403 (10.7)	209 (10.8)	194 (10.7)	0.792
Malaria rapid diagnostic test	268 (7.1)	131 (6.8)	137 (7.5)	0.362
Chest X-ray	255 (6.8)	131 (6.8)	124 (6.8)	0.950
Cerebrospinal fluid analysis	246 (6.6)	149 (7.7)	97 (5.3)	0.003
Glycated haemoglobin	231 (6.2)	136 (7)	95 (5.2)	0.044
Stool analysis	190 (5.1)	106 (5.5)	84 (4.6)	0.231
Lumbar puncture	175 (4.7)	114 (5.9)	61 (3.4)	0.000
Electrocardiogram	166 (4.4)	85 (4.4)	81 (4.5)	0.925
Echocardiography	166 (4.4)	93 (4.8)	73 (4)	0.681

*LAM* lipoarabinomannan.

### Inpatient outcomes

**Figure 2:**
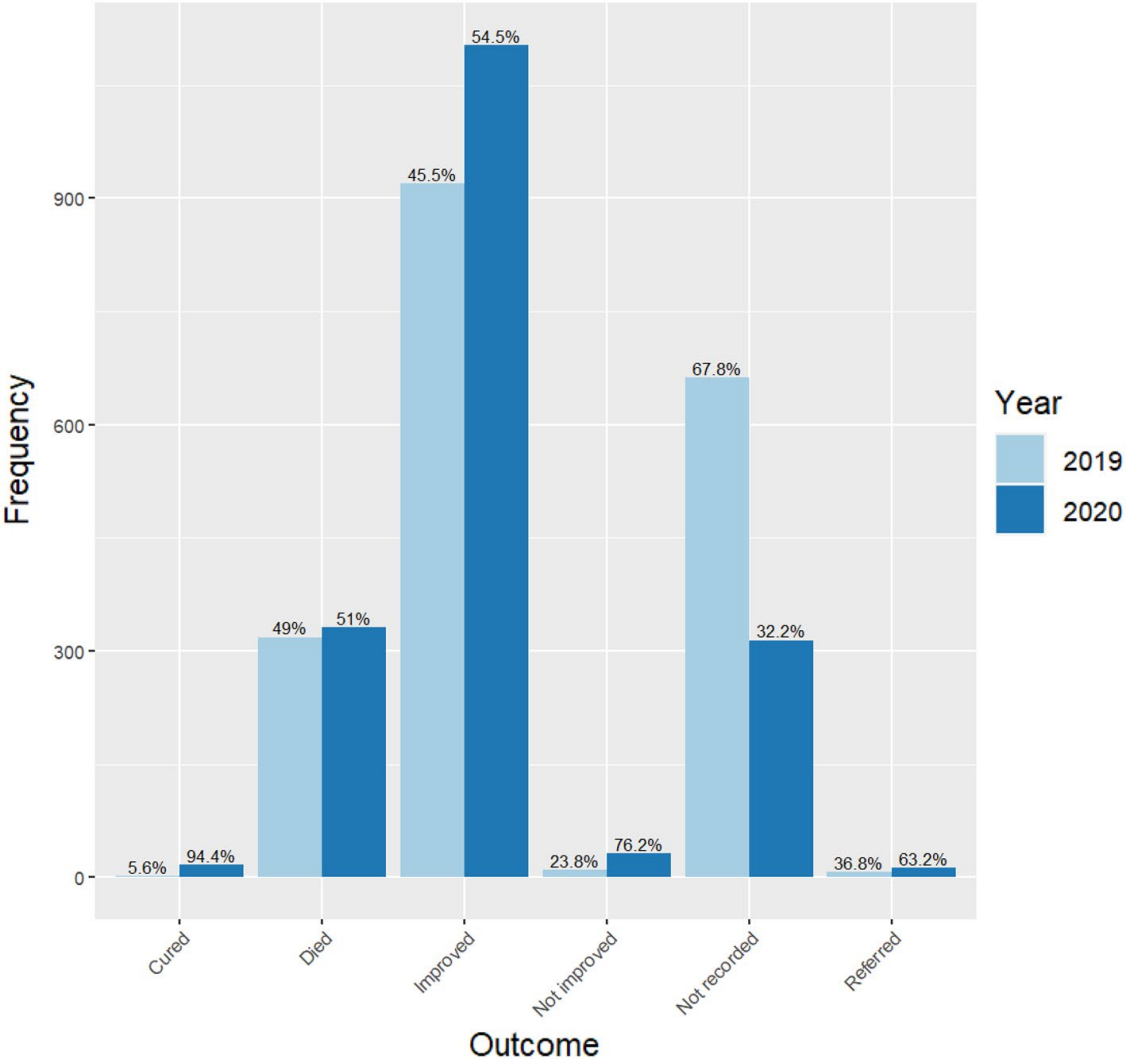
Vital outcomes of patients admitted at the in-patient department of Kiruddu National Referral Hospital between 2019 and 2020.

A total of 649 patients (17.3%, 95% confidence interval: 16.1–18.5%) died during the period of review (Fig. 2). The majority of those who died, referred or did not improve were admitted in the year 2020, although there was more missing data on the outcomes in this same year (Fig. 2). Crude mortality rates were higher in 2020 when compared to 2019 (18% vs 16%, p = 0.151). Table 4 shows the distribution of mortality across independent variables. Mortality was significantly associated with admission unit (p < 0.001), region of origin in Uganda (p = 0.028) and purpose of visit (respiratory symptom: p = 0.018, non-respiratory symptom: p = 0.001, referral status: p < 0.001, and others: p = 0.043) at bivariate analysis. Having HIV (p < 0.001), hypertension (p < 0.001), diabetes (p = 0.003), tuberculosis (p = 0.002), and asthma (p = 0.010). were similarly significantly associated with mortality at bivariate analysis.

**Table 4:** Distribution of mortality across social, demographic, and clinical characteristics.

Variables (N = 3749)	Died: n (%)	Alive: n (%)	p
Age in years			
13–17 years	0 (0)	18 (100)	0.143
18–35 years	243 (18)	1104 (82)	
36–59 years	271 (17.5)	1278 (82.5)	
60 + years	125 (15.9)	662 (84.1)	
Sex			
Male	321 (18.5)	1414 (81.5)	0.074
Female	328 (16.3)	1686 (83.7)	
Unit			
Infectious disease (female)	215 (21)	809 (79)	0.000
Infectious disease (male)	198 (22.2)	695 (77.8)	
Pulmonology	82 (17)	400 (83)	
Cardiology	94 (10.9)	770 (89.1)	
Endocrine	60 (12.9)	406 (87.1)	
Others	0 (0)	20 (100)	
Region of origin in Uganda			
Central	576 (17.5)	2717 (82.5)	0.028
East	21 (13.8)	131 (86.2)	
North	12 (32.4)	25 (67.6)	
West	28 (13.7)	176 (86.3)	
Religion			
Christian	439 (17.1)	2129 (82.9)	0.581
Muslim	111 (16.2)	574 (83.8)	
Patients with comorbidities			
HIV	364 (23)	1218 (77)	0.000
Hypertension	62 (9.2)	611 (90.8)	0.000
Diabetes Mellitus	81 (13.1)	536 (86.9)	0.003
Cardiac disorder	48 (15)	272 (85)	0.253
Tuberculosis	75 (23.5)	244 (76.5)	0.002
Asthma	7 (7.4)	87 (92.6)	0.010
Kidney disease	5 (12.2)	36 (87.8)	0.384
Malignancy	1 (5.3)	18 (94.7)	0.229
Chronic obstructive pulmonary disease	1 (5.3)	18 (94.7)	0.229
History of smoking	55 (15.2)	306 (84.8)	0.273
History of alcohol usage	142 (18.4)	630 (81.6)	0.372
Purpose of visits			
Non-respiratory symptom	639 (17.7)	2970 (82.3)	0.001
Respiratory symptom	398 (18.6)	1745 (81.4)	0.018
Referral	115 (23.4)	376 (76.6)	0.000
Need for oxygen	5 (22.7)	17 (77.3)	0.501
Others	40 (23)	134 (77)	0.043

### Factors associated with mortality

At simple logistic regression analysis (Table 5), patients admitted in the female infectious disease unit (IDF) (COR 1.8, 95% CI 1.3–2.5, p < 0.001) and male infectious disease unit (IDM) (COR 1.9, 95% CI 1.4–2.6, p < 0.001) wards were about twice more likely to die compared to those in the endocrine unit. Patients originating from the northern part of Uganda were also twice more likely to die (COR 2.3, 95% CI 1.1–4.5, p = 0.021 compared to those from central Uganda. Patients with HIV (COR 2.0, 95% CI 1.7–2.3, p < 0.001) and tuberculosis (COR 1.5, 95% CI 1.2–2.0, p = 0.002) were more likely to die, whereas those with hypertension (COR 0.4, 95% CI 0.3–0.6, p < 0.001), diabetes (COR 0.7, 95% CI 0.5–0.9, p = 0.003) and asthma (COR 0.4, 95% CI 0.2–0.8, p = 0.014) were less likely to die compared to those who did not have these comorbidities. Patients admitted due to non-respiratory symptoms were about thrice more likely to die compared to those with a respiratory complaint (COR 2.8, 95% CI 1.5–5.4, p = 0.019). Also, patients admitted as a referral were 1.6 times more likely to die than their non-referral counterparts (COR 1.6, 95% CI 1.2–2.0, p < 0.001).

**Table 5:** Factors associated with mortality in the in-patient department at Kiruddu National Referral Hospital, Uganda.

Variable (N = 3749)	Crude odds ratio (95% CI)	p-value	Adjusted odds ratio (95% CI)	p-value
Sociodemographic characteristics				
Age				
18–35 years	1.0		1.0	
35–59 years	1.0 (0.8–1.2)	0.702	1.1 (0.9–1.3)	0.380
60 + years	0.9 (0.7–1.1)	0.203	1.6 (1.2–2.1)	0.001
Sex				
Female	1.0			
Male	1.2 (1.0–1.4)	0.074	1.2 (0.9–1.5)	0.285
Admission unit				
Endocrine	1.0		1.0	
IDF	1.8 (1.3–2.5)	< 0.001	1.5 (0.9–2.4)	0.130
IDM	1.9 (1.4–2.6)	< 0.001	1.3 (0.8–2.2)	0.257
Pulmonology	1.4 (1.0–2.0)	0.075	1.1 (0.7–1.9)	0.587
Cardiology	0.8 (0.6–1.2)	0.278	0.8 (0.5–1.2)	0.277
Region of origin in Uganda				
Central	1.0		1.0	
East	0.8 (0.5–1.2)	0.243	0.9 (0.5–1.4)	0.510
North	2.3 (1.1–4.5)	0.021	2.1 (1.0–4.3)	0.045
West	0.8 (0.5–1.1)	0.169	0.8 (0.5–1.2)	0.306
Religion				
Christian	1.0			
Muslim	1 (0.8–1.3)	0.581		
Period of admission				
Before COVID-19 pandemic (2019)	1.0		1.0	
During COVID-19 pandemic (2020)	1.1 (1.0–1.3)	0.151	1.2 (1.0–1.5)	0.018
Comorbidities				
HIV/AIDS	2.0 (1.7–2.3)	0.000	1.5 (1.2–1.9)	< 0.001
Hypertension	0.4 (0.3–0.6)	0.000	0.5 (0.4–0.7)	< 0.001
Diabetes	0.7 (0.5–0.9)	0.003	1.1 (0.8–1.7)	0.494
Tuberculosis	1.5 (1.2–2.0)	0.002	1.1 (0.8–1.5)	0.478
Reason for admission				
Referral	2.8 (1.5–5.4)	0.002	1.5 (1.2–1.9)	< 0.001
Respiratory symptom	1.2 (1.0–1.5)	0.019	1.5 (1.3–1.9)	< 0.001
Non-respiratory symptom	1.6 (1.2–2.0)	0.000	3.4 (1.6–7.1)	0.001
Need for oxygen	1.4 (0.5–3.8)	0.503	1.6 (0.6–4.5)	0.392

In the final multivariable logistic regression model (Table 5), only age, period of admission, comorbidities (HIV and hypertension), and purpose of visits remained significantly associated with mortality. Age 60 years and above (AOR 1.6, 95% CI 1.2–2.1, p = 0.001), patients admitted during the COVID-19 pandemic (2020) (AOR 1.2, 95% CI 1.04–1.5, p = 018), those with HIV (AOR 1.5, 95% CI 1.2–1.9, p < 0.001), and those admitted as a referral from peripheral facilities (AOR 1.5, 95% CI 1.2–1.9, p < 0.001) were all significantly more likely to die. Conversely, hypertension remained significantly associated with less mortality compared to those without (AOR 0.5, 95% CI 0.4–0.7, p < 0.001).

## Discussion

The COVID-19 pandemics has had a devastating impact on health care systems across the globe with significant reduction in overall service utilization (37% reduction), clinic visits (42%), admissions (28%), utilization of diagnostics (31%) and therapeutics (30%)^7^. In this study, we compared inpatient attendance, diagnostic tests performed on inpatients, patient characteristics and inpatient mortality between January–July 2019 (before the COVID-19 pandemic) and January–July 2020 (during the COVID- 19 pandemic) at an urban tertiary facility in Uganda. We found that inpatient admissions declined by 6.1% in 2020. People with respiratory conditions were less likely to be admitted during the pandemic at our study site although there were more COPD admissions. Additionally, there were fewer laboratory tests performed in 2020 and this was particularly concerning for sputum GeneXpert, blood smears for malaria, blood sugar, and glycated hemoglobin. While the difference in the crude mortality was not statistically significant (18% in 2020 vs. 16% in 2019, p = 0.15), being admitted during the pandemic was associated with 30% higher odds of mortality at multivariable analysis.

A decline in the number of inpatient admissions during the COVID-19 pandemic has been widely reported, although reports from Uganda a few. A decline in hospitalization among children with sickle cell disease was noted at an urban referral facility in Uganda during the pandemic^13^. A recent study found a decline in admission on adult medical wards in Kenya from April to June 2020 that coincided with national movement restrictions to curb the spread of COVID-19^14^. The decline in admissions was modest in our study compared to that reported in other studies in Croatia (21%)^15^, China (26%)^16^, Belgium (29–39%)^17^ and Spain (16.8%)^18^. Clearly, these countries had a larger scale of the pandemic and instituted more stringent lockdowns. Nonetheless, the reduction in admissions is likely due to lack of access to the hospital during the pandemic because of the transport restrictions that were instituted by government^19, 20^. This is supported by our data which shows fewer admission in 2020 for people from northern Uganda, the furthest region from the hospital. However, we cannot rule out that people stayed away from hospitals due to fear of infection or that referring clinicians had a higher threshold for referral to our facility during the pandemic period^21^.

Similar to our study, a large study in US hospitals reported a decline in admissions among patients with non-COVID related respiratory conditions^22^. A similar observation was made in South Africa^23^ and Spain^18^. While the reason for this is not apparent, we suppose that people with respiratory symptoms tended to shy away from hospitals to avoid stigma since many respiratory conditions have similar symptoms to COVID-19. Another possible explanation is that the lockdown resulted in lower pollution rates in Uganda and this coupled with use of face coverings could have led to a lower incidence of respiratory complaints^24, 25^. Additionally, Uganda had dedicated COVID treatment units in Kampala. Therefore, patients with respiratory symptoms could have been preferentially referred to those centers. Paradoxically, more people with COPD were admitted during the pandemic in our study. This could point to lack of access to the weekly respiratory outpatient clinic services that were closed during the pandemic. As such, the pandemic could have affected access and/or adherence to medicines for patients in chronic care. About 36% of people with chronic illnesses could not access medicines during the lock down s^26^.Conversely, one study in Spain reported that the rate of COPD exacerbations was lower during the pandemic than before although their sample size was small and they enrolled patients who were classified as frequent exacerbators^27^.

We observed a > 20% decline in the number of diagnostic tests performed including malaria (thick blood smears), tuberculosis (GeneXpert) and diabetes (blood sugar and glycated hemoglobin). This is worrisome and potentially affected TB and malaria detection, monitoring of diabetes mellitus treatment and overall treatment of patient who might have had these diseases. The decline in the number of tests performed is not proportionate to the decline in the number of inpatients in this study. Therefore, we cannot attribute the decline in the tests solely to the overall decline in inpatient numbers. Reported TB cases declined by 43% during the COVID lock down from pre-pandemic period in Uganda and this was estimated to increase TB-related mortality by 14%^28^. In rural Uganda, a recent study reported a decline in the number of malaria rapid diagnostic tests and the number of people initiating anti-malarial treatment before and during the pandemic^29^. Disruption of screening services for cardiovascular disease, such as diabetes mellitus, at health facilities has also been reported in Uganda during the pandemic^30^. Similar declines in laboratory test volumes (including the HbA1c) during the pandemic were noted elsewhere; and partial recovery to pre-pandemic levels might affect long term monitoring of people with chronic diseases^31, 32^. Taken together, the decline in these diagnostic tests due to the pandemic could have lasting effect on elimination of TB and malaria^33, 34^.

We found that being admitted during the pandemic was associated with higher odds of mortality. This is possibly because of delayed referrals or referral bias of critically ill individuals. This is supported by the higher likelihood of death we observed among referred patients. Additionally, clinical teams might have found difficulty in making diagnoses for life-threatening conditions such as sepsis, bacterial meningitis, acute kidney injury, acute liver injury and electrolyte abnormalities. From our study, there was a decline in the number of tests performed for these conditions; that is, fewer cerebral spinal fluid analyses, urinalysis, full blood count, serum electrolytes, serum creatinine and liver function tests. There is scanty data from medical wards in Uganda to compare our findings to. However, a study at a newborn unit in Uganda reported a 14% decline in admissions, an increase in inpatient mortality (16% vs. 11%) and patients referred from other facilities had a 55% higher mortality^35^. A large study of more than 32 million admissions in the US also showed a 30% increase in all-cause mortality among inpatients during the pandemic period^36^. A similar observation was made among non-COVID-19 admissions at 4626 US hospitals^37^. Conversely, a large study in Germany reported lower risk for all-cause mortality during the pandemic period when people with SARS-CoV-2 were excluded in the analysis^38^. However, their population had significantly low HIV infection rates compared to our study where 1 in 4 people had HIV infection. Moreover, HIV was independently associated with mortality in our study, and alongside advanced age, is consistently observed to increase the risk of mortality on medical wards in Uganda^39^.

Our study is limited in several ways. First, this was a single center study and this limits generalizability of findings. Moreover, the study site is a tertiary hospital, and this could introduce referral bias whereby critically ill individuals were referred during the pandemic. Thirdly, we did not evaluate system factors such as stock out of supplies and health worker absenteeism during the pandemic that could potentially affect the volume of diagnostic tests performed and the overall patient outcomes. For 26% of the participants included, the outcome was not documented in the files. We modelled these as non-fatal outcomes since death is meticulously documented in our hospital and admission units conduct mortality audits. Nonetheless, this might have introduced some misclassification bias. Our study included the months of January and February 2020 in the COVID pandemic period yet COVID-19 was declared a pandemic in March 2020. This could have affected the accuracy of our estimates. However, we believe that perceptions and health care utilization were already affected in the two months preceding March when cases and deaths started to be reported outside China and human-to-human transmission was confirmed^40^. Lastly, data on the COVID-19 vaccination status were not available in the files. Therefore, we were unable to determine if the COVID-19 vaccine affected the outcomes.

## Conclusion

There was a modest decline in the number of admissions during the COVID-19 pandemic at a tertiary hospital in Uganda. There was a decline in the diagnostic tests during the pandemic and this could affect gains made in the control and elimination of TB and malaria. Being admitted during the pandemic was associated with 30% higher odds of mortality. Policy makers in Uganda need to build resilience in health systems to deal with future pandemics.

## Data Availability

Datasets used in this analysis are available from the corresponding author on reasonable request.
